# A Case of Cardiogenic Stroke With a Novel LMNA Variant (c. 1135C>A; p.Leu379Ile)

**DOI:** 10.7759/cureus.37824

**Published:** 2023-04-19

**Authors:** Naoki Tokuda, Yukiko Tsuji, Michio Inoue, Ichizo Nishino, Masahiro Makino

**Affiliations:** 1 Department of Neurology, Kyoto Okamoto Memorial Hospital, Kyoto, JPN; 2 Department of Neurology, Japanese Red Cross Kyoto Daini Hospital, Kyoto, JPN; 3 Department of Neuromuscular Research, National Institute of Neuroscience, National Center of Neurology and Psychiatry, Tokyo, JPN

**Keywords:** lmna gene, emery–dreifuss muscular dystrophy, laminopathy, mechanical thrombectomy, intravenous tissue plasminogen activator, ischemic stroke

## Abstract

Laminopathy is muscular dystrophy caused by an *LMNA* gene mutation. It is characterized by cardiac disease such as atrial fibrillation. We report a case of laminopathy in a 49-year-old woman who presented with cardiogenic stroke. She had experienced weakness in her limb-girdle muscles since childhood, atrial fibrillation, cardiomyopathy, and mild contracture of the ankle joints, and had a familial history of heart disease. Gene analysis identified a novel heterozygous variant, c. 1135C>A (p.Leu379Ile), in the *LMNA* gene. Laminopathy can be an underlying disease in ischemic stroke, especially in young to middle age.

## Introduction

Laminopathy is a rare genetic disorder that is caused by mutations in the *LMNA* gene, which encodes for proteins of the nuclear lamina. Laminopathy is characterized by slowly progressive muscle weakness with early-onset contractures and cardiac conduction disturbance [1,2]. In particular, atrial fibrillation is a crucial risk factor for cardiogenic embolism. We report a Japanese patient who was diagnosed with laminopathy due to a novel *LMNA* gene variant, after experiencing a cardiogenic stroke caused by atrial fibrillation.

## Case presentation

**Figure 1:**
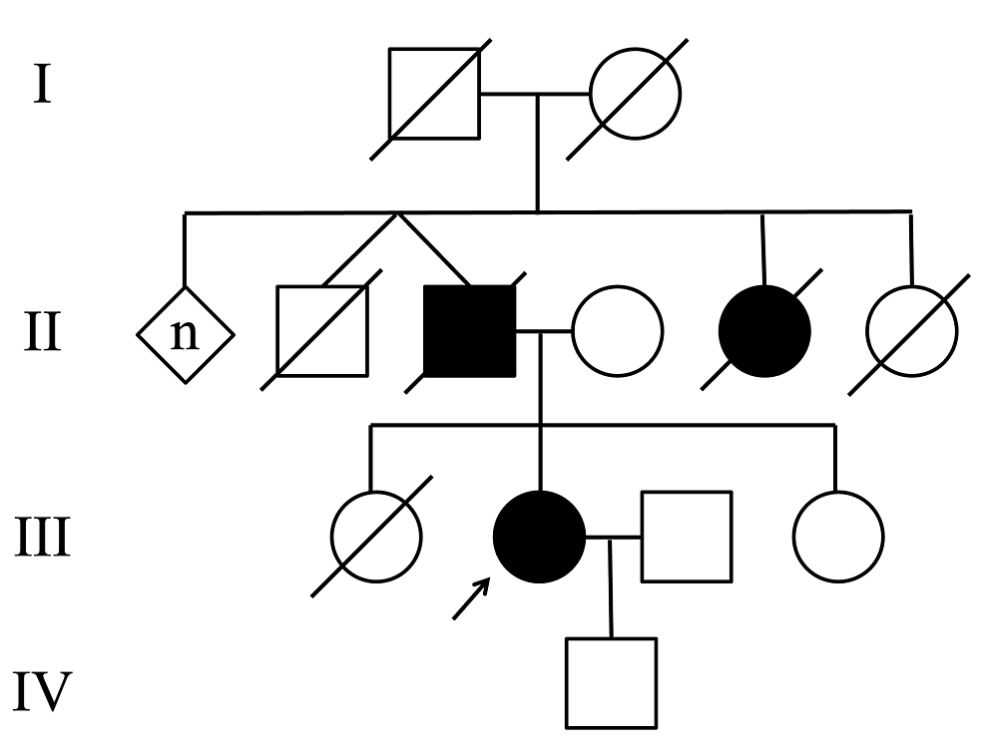
The family pedigree. A square indicates a male individual; a circle indicates a female individual; a rhombus indicates unknown sex; a diagonal line indicates a deceased individual; n indicates that the number is unknown; black-filled square or circle indicates male or female individual, respectively, with muscular and cardiac symptoms.

A 49-year-old woman, who had had difficulty walking since childhood but had not undergone neurologic or cardiologic examinations, was transferred to the emergency room 2.5 hours after developing the symptom of sudden-onset left-side weakness. The father of the patient reportedly had a gait disturbance and cardiac pacemaker implantation. However, his medical history was not fully accessed because he was already deceased and had died in his 70s. The twin brother of the patient’s father had died in childhood for unknown reasons. The paternal aunt had heart disease and had died in her 70s. The patient had a healthy son with neither gait disturbance nor heart disease (Figure 1).

**Figure 2:**
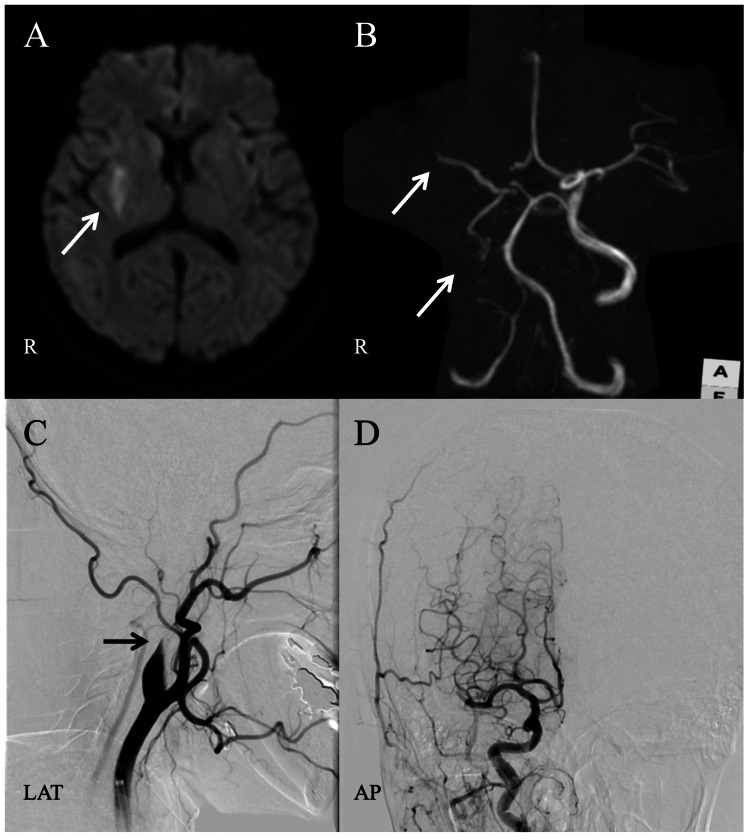
Cerebral imaging findings in this case. (A) Brain magnetic resonance imaging findings. The diffusion-weighted image on admission shows an infarct in the right hemisphere. (B) Magnetic resonance angiography on admission shows a right internal carotid artery occlusion. (C) The right carotid artery angiogram shows an occlusion at the cervical internal carotid artery. (D) After mechanical thrombectomy, the right internal carotid artery was partially recanalized. LAT, lateral view; AP, anterior-posterior view

On arrival at the emergency room, her blood pressure was 191/124 mmHg, and heart rate was 120 beats/minute with atrial fibrillation with rapid ventricular response. She had left hemiparesis and mild paralytic dysarthria, but she did not have impaired consciousness or any sign of aphasia. Her score was 7 on the National Institutes of Health Stroke Scale (NIHSS) score. The non-contrast head computed tomography (CT) scan revealed no evidence of intracranial hemorrhage or significant acute ischemic changes. Brain magnetic resonance imaging (MRI) showed an acute ischemic stroke of the right putamen and corona radiate (Figure 2A). Magnetic resonance angiography showed occlusion of the right internal carotid artery and the right middle cerebral artery (Figure 2B). We treated the patient with intravenous tissue plasminogen activator and mechanical thrombectomy with a stent retriever, which resulted in a score of 2a on the modified Thrombolysis in Cerebral Infarction scale (Figures 2C, 2D). Her neurological symptoms, including left hemiplegia, improved dramatically.

On the fifth hospital day, a neurological examination of the patient revealed that the cranial nervous system findings were within normal limits, muscle atrophy of the proximal extremities, and low grip power of 15 kg in the right hand and 11 kg in the left hand. The manual muscle test revealed grade 3 weakness (based on the Medical Research Council scale) in the iliopsoas muscles and the muscles of the proximal arms, and grade 4 weakness in the other proximal muscles of the lower limbs, except for the iliopsoas muscles, with no difference between the right and left sides. The distal muscles of the upper and lower limbs had normal strength. A rigid spine was not apparent, but slight contracture of the ankle joint was observed. The sensation of all modalities was preserved. Tendon reflexes in all extremities were reduced. She had the Trendelenburg gait on walking. The Mini-mental State Examination and Frontal Assessment Battery scores were 29/30 and 13/18, respectively. Her NIHSS score was zero.

**Figure 3:**
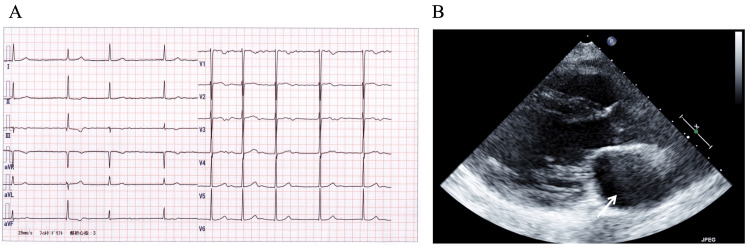
Cardiac imaging findings in this case. (A) An electrocardiography on the fifth day of hospitalization showed atrial fibrillation. (B) Transthoracic echocardiography revealed left atrium enlargement.

**Figure 4:**
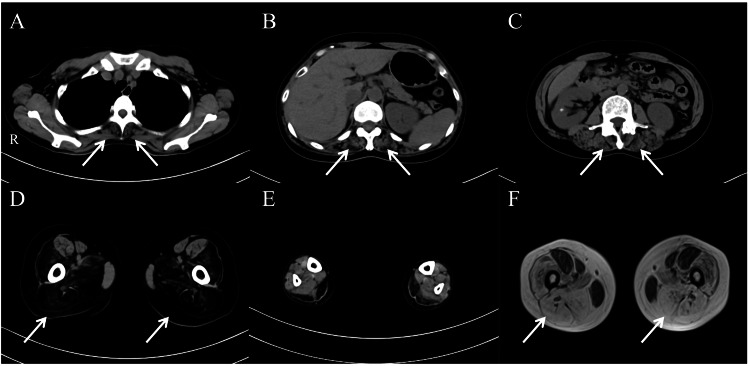
Muscle imaging findings in this case. (A-E) Muscle computed tomography findings. (F) Muscle magnetic resonance T2-weighted image at the thigh level (i.e., same level as in image D). The patient presents with atrophy of the paraspinal and dorsal thigh muscles and fat displacement of the thigh muscles, except for the rectus femoris, sartorius, and gracilis muscles.

Laboratory test findings revealed that the brain natriuretic peptide level was elevated at 126.2 pg/mL, whereas the creatinine kinase and D-dimer levels were within the normal range at 159 IU/L and 0.4 µg/mL, respectively. Electrocardiography revealed atrial fibrillation and no apparent ST-segment changes or pause (Figure 3A). Transthoracic echocardiography revealed left ventricular wall diffuse hypokinesis (i.e., left ventricular ejection fraction of 49%) and left atrium enlargement (i.e., left atrial volume index of 60 mL/m2) (Figure 3B). The results of the nerve conduction study were normal. A needle electromyogram and muscle biopsy were not performed due to a lack of patient consent. On muscle imaging (i.e., whole-body computed tomography (Figures 4A-4E) and axial thigh MRI (Figure 4F)), the most remarkable change was near-complete fat replacement of the thigh muscles - except for the rectus femoris, sartorius, and gracilis muscles, which were hypertrophied, possibly via a compensatory mechanism-and the gastrocnemius muscles. In addition, the paraspinal muscles were atrophic. Cardiac MRI revealed no abnormal findings.

We started anticoagulation therapy without catheter ablation or implantation of a pacemaker or cardiac defibrillator, while consulting with cardiologists. She was transferred to a rehabilitation facility on the 19th day of admission with mild gait disturbance (modified Rankin scale 1).

On account of the limb-girdle pattern muscular weakness and atrophy, atrial fibrillation, cardiomyopathy, and cardiac disease with an autosomal dominant inheritance, we strongly suspected *LMNA*-related muscular dystrophy. After obtaining written informed consent from the patient, deoxyribonucleic acid (DNA) was extracted from the patient’s lymphocytes. Genetic analysis was conducted, using the target gene panel with the next-generation sequencer Ion Personal Genome MachineR System (Thermo Fisher Scientific, Waltham, MA, USA), as previously described [3]. The analyzed genes of known muscular dystrophies are as follows: *ARGN*, *ALG13*, *ANO5*, *B3GALNT2*, *G3GNT1,* *CAPN3*, *CAV3*, *CHKB*, *COL12A1*, *COL6A1*, *COL6A2*, *COL6A3*, *DAG1*, *DES*, *DMD*, *DNAJB6*, *DOK7*, *DOLK*, *DPAGT1*, *DPM1*, *DPM2*, *DPM3*, *DYSF*, *EMD*, *FAT1*, *FHL1*, *FKRP*, *FKTN*, *FLNC*, *GFPT1*, *GMPPB*, *ISPD*, *ITGA7*, *KLHL9*, *LAMA2*, *LARGE*, *LMNA*, *MEGF10*, *MICU1*, *MYOT*, *PLEC*, *POMGNT1*, *POMGNT2*, *POMT1*, *POMT2*, *PTRF*, *SGCA*, *SGCB*, *SGCD*, *SGCG*, *POMK*, *SMCHD1*, *STIM1*, *SYNE1*, *SYNE2*, *TCAP*, *TMEM43*, *TMEM5*, *TNPO3*, *TRAPPC11*, and *TRIM32*. Variants with allele frequencies less than 0.01 in exonic and splicing regions were extracted and examined for disease-causing mutations. The aforementioned gene analysis was approved by the Institutional Review Boards of the National Center of Neurology and Psychiatry (Tokyo, Japan). We found a previously unreported heterozygous variant, c.1135C>A, in the *LMNA* gene in which leucine is changed to isoleucine at position 379. This variant was confirmed with Sanger sequencing. Therefore, we finally diagnosed her with laminopathy, which was consistent with her clinical features. Other family members did not undergo genetic analyses because informed consent was not obtained.

## Discussion

The *LMNA* gene encodes lamin A and lamin C, which are components of the nuclear envelope protein and are expressed in a wide range of tissues, including heart and skeletal muscle [4]. *LMNA* gene mutations are associated with widely varying phenotypes such as autosomal dominant Emery-Dreifuss muscular dystrophy (EDMD), autosomal dominant dilated cardiomyopathy with conduction defects, Charcot-Marie-Tooth disease type 2B1, familial partial lipodystrophy, Hutchinson-Gilford progeria syndrome, and atypical Werner’s syndrome, which are generally considered laminopathies [2]. Limb-girdle muscular dystrophy (LGMD) is also a phenotype of laminopathy known as LGMD1B. However, at the European Neuromuscular Centre International Workshop in 2018, it was classified as a form of EDMD and excluded from the LGMD group [5]. The variant detected in this patient, p.L379I in the *LMNA* gene, has not been previously reported, whereas other amino acid substitution mutations at the same site (p.L379F and p.L379V) exhibit the phenotypes of LGMD [6] and dilated cardiomyopathy with conduction defects [7], respectively. Based on the American College of Medical Genetics (ACMG) classification, the variant in this patient had three moderate evidence (Moderate evidence of pathogenicity (PM)1, PM2 and PM5) and two supporting evidence (supporting evidence of pathogenicity (PP)3 and PP4), which suggested that the findings of this variant were consistent with “likely pathogenic” [8].

Autosomal dominant EDMD is characterized by early-onset elbow and Achilles tendon contracture, weakness in humeroperoneal muscles, cardiac conduction defects, and cardiomyopathy. In addition, scoliosis and rigid spine are often observed because of paraspinal muscle involvement [1, 2]. In laminopathy, the vastus lateralis is often the most severely affected, while the medial thigh muscles are usually less affected. However, the involvement of the thigh muscles is variable [9]. Clinically, muscle symptoms may also be of the limb-girdle type, which was previously called LGMD 1B [10]. In the present case, joint contracture was slightly detectable in the ankles, and the distribution of muscle symptoms was not the humeroperoneal type but the limb-girdle type. As described previously, the phenotype of laminopathy is variable, and an early diagnosis can be challenging.

The *LMNA* mutation per se has been independently associated with thromboembolism after adjustment for possible confounders, including atrial fibrillation and left ventricular ejection fraction [11]. In this report, *LMNA* mutation carriers had altered platelet function and increased thrombin generation, thereby promoting thrombogenicity. Furthermore, among *LMNA* mutation carriers, atrial fibrillation per se carries nine-fold increased odds for ischemic stroke [12]. In the present case, even if atrial fibrillation had been detected before the stroke, the CHA_2_DS_2_-VASc score would have been one and anticoagulation therapy may not have been indicated [13]. However, if laminopathy had been diagnosed, it may have been reasonable to initiate anticoagulation therapy, despite the lack of specific guidelines or recommendations for laminopathy without atrial fibrillation. This could have potentially prevented stroke. Future studies are needed to clarify the indication for anticoagulation therapy in laminopathy without atrial fibrillation. It is important for clinicians to be aware that laminopathy is an underlying disease of cardiogenic embolism, especially in young to middle-aged patients.

We have successfully treated ischemic stroke in patients with laminopathy by using intravenous tissue plasminogen activator and mechanical thrombectomy with good neurological outcomes. Intravenous tissue plasminogen activator [14] and mechanical thrombectomy have been reported for cerebral infarction caused by laminopathy [15]. Hyperacute treatment for cerebral infarction caused by laminopathy is expected to be as effective as that for typical cerebral infarction. After the acute phase, patients with laminopathy should be carefully monitored in cooperation with cardiologists with regard to anticoagulation therapy, arrhythmia treatments such as pacemaker and implantable cardioverter-defibrillator, and heart failure management [12].

## Conclusions

We present a case of laminopathy with a new genetic mutation that was diagnosed after a cardiogenic stroke. In this case, laminopathy had not been diagnosed before the stroke.

Laminopathy should be considered as a potential cause of cardiogenic stroke, particularly in young to middle-aged patients with a family history of heart or muscle disease, or muscle symptoms. Diagnosing laminopathy is crucial, as appropriate anticoagulation therapy and device implantation for lethal arrhythmias may significantly improve the prognosis.
